# Supplementary material for: Observation of solid–solid transitions in 3D crystals of colloidal superballs
Source: Nat Commun. 2017 Feb 10;8:14352. doi: 10.1038/ncomms14352 (PMC5309858; doi:10.1038/ncomms14352)
Supplement: Supplementary Information — Supplementary Figures, Supplementary Table, Supplementary Note and Supplementary References. [file ncomms14352-s1.pdf]

Supplementary Figures

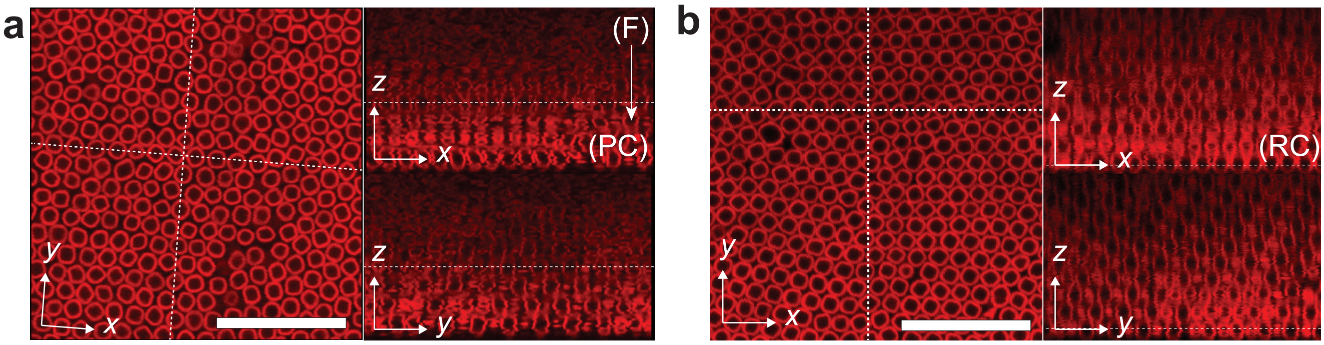

Supplementary Figure 1. 3D structure of the crystalline phases formed by superballs. Confocal laser scanning microscopy images of *x*-*y*-*z* stacks of fluorescently labelled hollow silica superballs with $m = 3.0$ just above the glass wall of a) a plastic crystal (PC) forming from a fluid (F) at the top after 2 hours and b) a fully formed rhombohedral crystal (RC) obtained after 20 hours. The layering on the glass wall as well as the stacking of the layers can be seen in the *x*-*z* and *y*-*z* views. Due to the refractive index mismatch between ethanol and silica the signal of the superballs decreases significantly above z ~20 µm and we cannot observe the bulk structure of the superball sediments. Scale bars are 10µm.

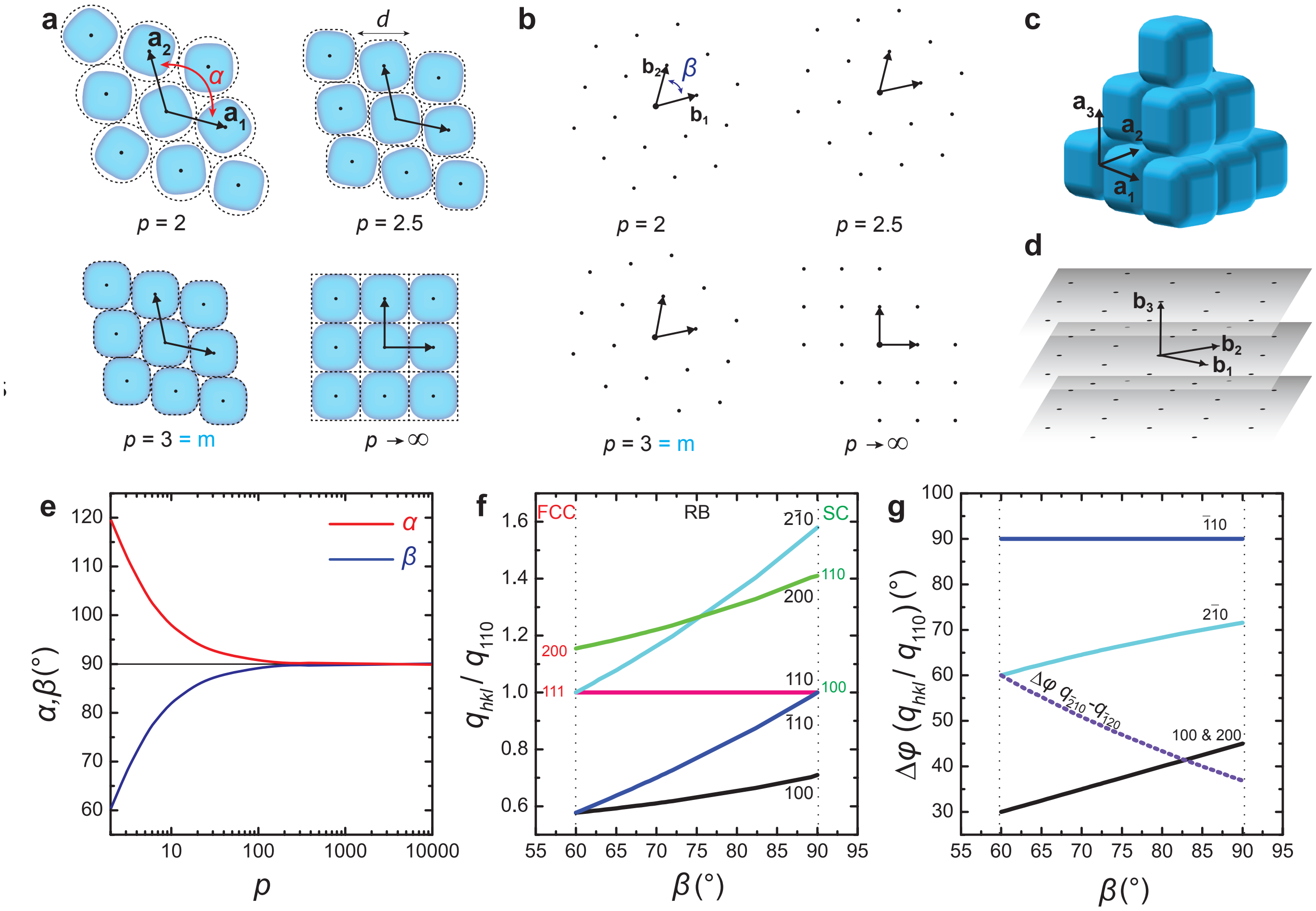

Supplementary Figure 2. Structural characteristics in real and reciprocal space. a) Illustration of the angle variable rhombohedral real-space lattice described by real space vectors $\mathbf{a_1}$ and $\mathbf{a_2}$, with angle α, and the lattice-site shape parameter, p, and lattice spacing along the superball face-to-face direction, d. In this case the lattice sites are occupied by superball particles with shape parameter $m = 3$, resulting in a close packed structure when $p = m$ and hence $d = L$. b) Illustration of the corresponding reciprocal space RC lattice described by reciprocal space vectors $\mathbf{b_1}$ and $\mathbf{b_2}$ with angle, β. c) Schematic illustration of the 3D lattice in real space consisting of stacked rhombohedral planes spaced by real space vector $\mathbf{a_3}$. d) Resulting reciprocal space lattice that consists of planes of *hkl* peaks spaced by reciprocal space vector $\mathbf{b_3}$. e) Real-space lattice angle, α, and reciprocal space lattice angle, β, as a function of lattice shape parameter p. f) Calculated ratios of diffraction peak positions of the in-plane reflections of the rhombohedral crystal (RC) as a function of β. Together with the *hkl* indices of the face centered cubic (FCC) and simple-cubic (SC) planes indicated at 60° and 90°, respectively. g) Calculated angle $\Delta\varphi$ between the q_{hkl} and the q_{110} peaks.

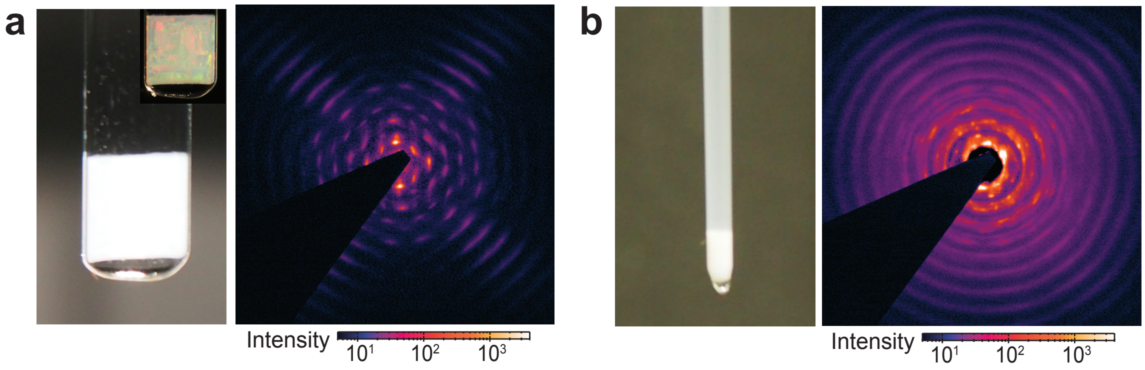

Supplementary Figure 3. The effect of capillary shape on the poly-crystallinity of the superball sediments. a) Photograph of the sediment formed by superballs with m = 3.6 in water in a rectangular capillary under white light illumination (inset: Illumination from the back of the capillary, due to the size of the superball particles optical Bragg reflections are only visible under this geometry) together with 2D μrad-SAXS pattern obtained in the bulk of the sediment, showing the formation of a mono-crystalline structure. b) Photograph of the same sediment now formed in a round capillary with internal diameter 1 mm. Together with the 2D μrad-SAXS pattern obtained in the bulk showing the formation of a poly-crystalline structure. Besides the difference in long-range order of the crystals the different form factor ($P(\mathbf{q})$) contributions in the pattern also show the orientations of the superball particles. Therefore, we conclude that wall anchoring of the superballs and subsequent alignment of the crystal planes are the cause of the mono-crystalline structures.

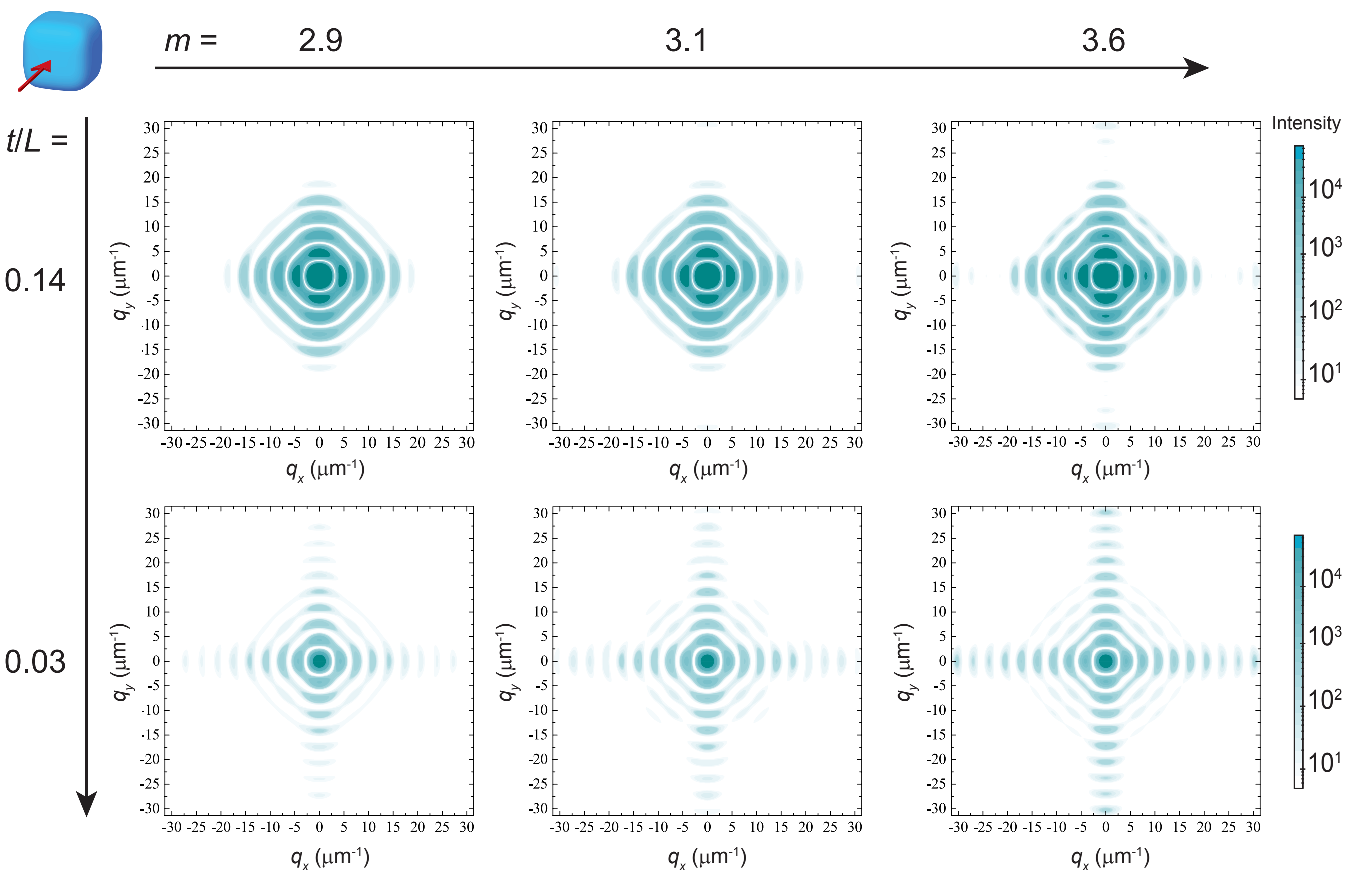

Supplementary Figure 4. The effect of the hollow superball shell properties on the fine structure of the anisotropic form factor. Numerically calculated 2D patterns of the form factor, $P(\mathbf{q})$, with different shape parameters m and different relative shell thickness t/L for a hollow superball shell a oriented with one of its faces perpendicular to the x-ray beam (schematically shown in top left corner). Slight differences in shape parameter m change the anisotropy of the pattern along the azimuthal φ direction, while the shell thickness influences the q-range of the horizontal and perpendicular fringes that originate from the superball faces.

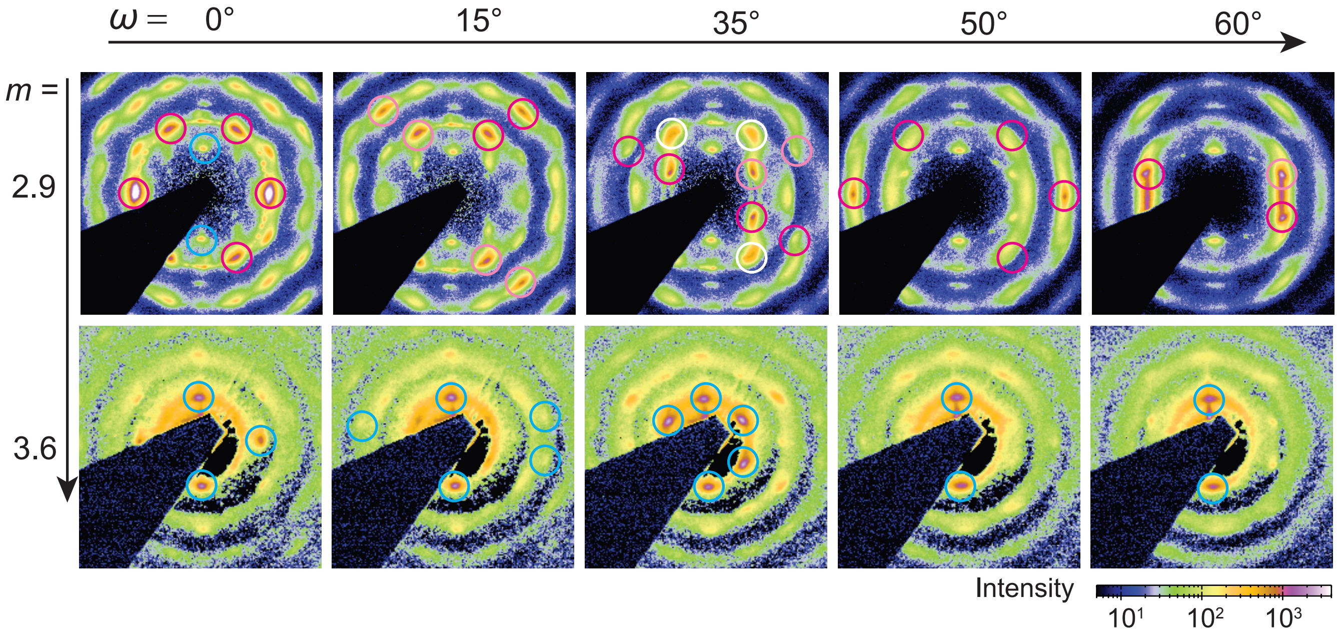

Supplementary Figure 5. **Rotational scans of superball crystals.** Selected 2D µrad-SAXS patterns obtained at different angles, ω, of the crystalline sediments of two superball particles with m = 2.9 and m = 3.6. The different diffraction peaks in the patterns are related to the two different stacking sequences of the rhombic lattice planes. Scattering peaks originating from hollow-site stacking (HRC) are indicated with pink circles and bridge-site stacking (BRC) with blue circles. Due to the presence of two hollow-sites in a RC plane, twinned crystal structures are formed resulting in symmetric patterns (light pink circles). For m = 2.9 the patterns show dominant HRC but also some BRC is present. In addition, the diffraction peaks at ω = 35° indicated with the white circles can only be obtained in a structure with a combination of hollow-site and bridge-site stacking of the rhombohedral lattice planes, as these diffraction peaks are not allowed in either one of the crystal structures. For m = 3.6 the structure is clearly BRC.

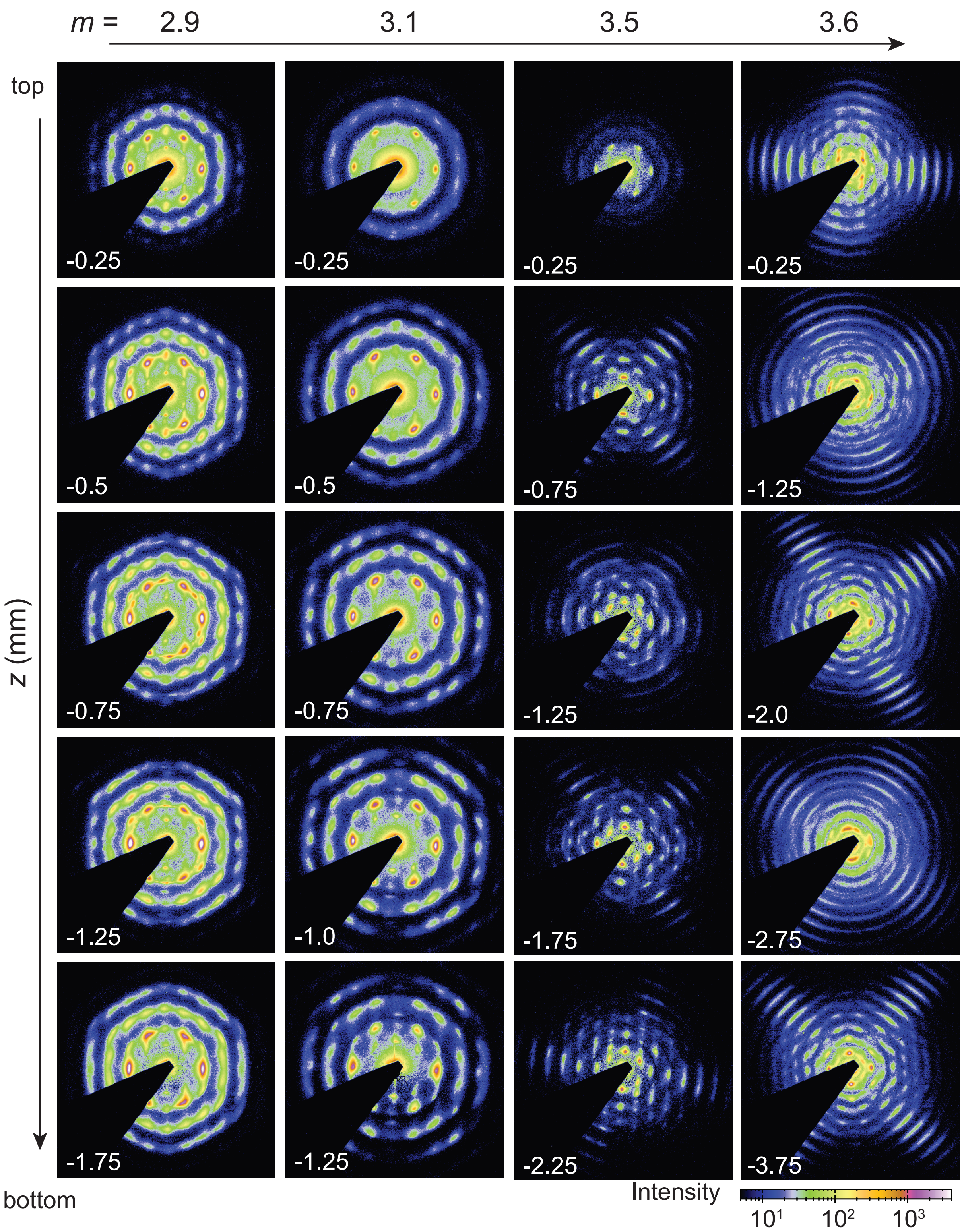

Supplementary Figure 6. Height scans of crystalline superball sediments. Selected 2D µrad-SAXS patterns obtained at different heights *z* (shown in left bottom corner) in the sediments of the differently shaped superball colloids. A transition in the 2D patterns from top to bottom indicates the presence of a structural transition with increased pressure.

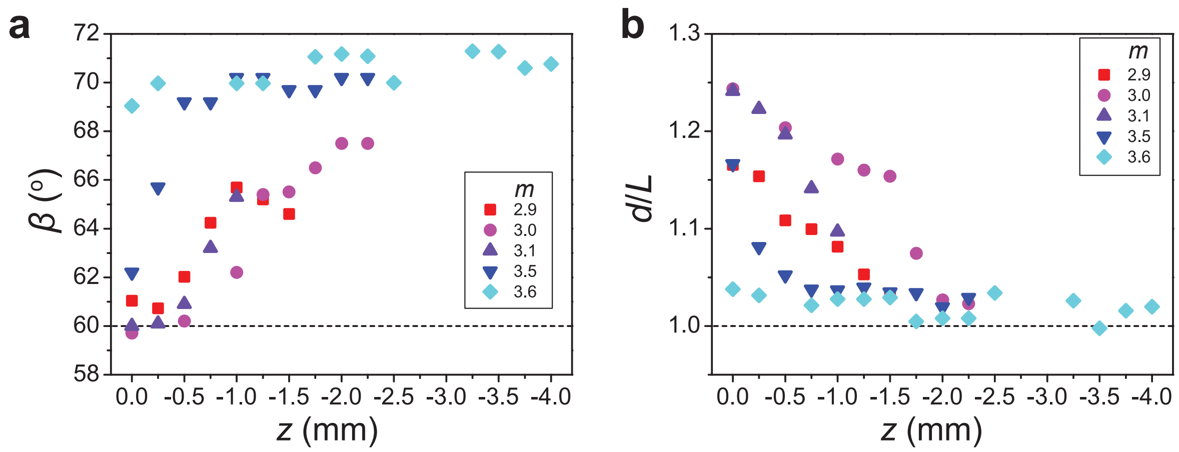

Supplementary Figure 7. Structural analysis of the full superball sediments. Structural angle β and lattice spacing to superball edge-to-edge length ratio, d/L, for each superball shape extracted from the positions of the *hkl* peaks in each 2D μrad-SAXS pattern obtained at different heights in the sediments.

Supplementary Table 1. Summary of the superball particle characteristics. The edge-to-edge-length, L, and shell thickness, t, were determined from statistical analysis of the TEM micrographs of the composite hematite silica superballs. Using a contour fit of the 2D projection of the hollow silica superballs the exact superball shape expressed in the shape parameter m was determined[1,2]. The gravitational length of the particles, L_g, was estimated by assuming a perfect hollow silica cube with L and t of the respective superballs according to: $L_g = \frac{kT}{(L^3-(L-2t)^3)\Delta\rho g}$, where kT is the thermal energy, g is the gravitational acceleration and $\Delta\rho = \rho_p - \rho_s$, with ρ_s the density of the respective solvent at room temperature ($T = 293$ K) and ρ_p the density of the amorphous silica that is estimated as $\rho_p = 1.6$ g cm^{-3}.

**Composition**	***L* (nm)**	**σ*L* (nm)**	***t* (nm)**	***m***	***t*/*L***	**Solvent**	**L_g (nm)**
**Silica**	774	35	108	2.9	0.14	H_2O	2372
**Fluorescent Silica**	1261	45	40	3.0	0.03	EtOH	1421
**Fluorescent silica**	1273	51	46	3.0	0.04	EtOH	1200
**Silica**	651	46	59	3.1	0.09	H_2O	5524
**Silica**	1033	45	50	3.5	0.05	H_2O	2370
**Silica**	1266	27	43	3.6	0.03	H_2O	1781

Supplementary Note 1:

Definition of the real and reciprocal space vectors of the angle-variable rhombohedral crystals.

In order to describe the full structural transition from a plastic FCC crystal to a rhombic crystal (RC) as well as the two observed stacking sequences with the same *hkl* indices we use the following real and reciprocal space vectors. The angle variable rhombohedral lattice planes are described by the real-space basis lattice vectors $\mathbf{a}_1$ and $\mathbf{a}_2$ with angle, α, that produces the reciprocal space vectors $\mathbf{b}_1$ and $\mathbf{b}_2$ with angle, β, as shown in Supplementary Figure 2a and Figure 2b. The vectors $\mathbf{a}_1$ and $\mathbf{a}_2$ are related to the Λ_1-lattice vectors $\mathbf{e}_1$ and $\mathbf{e}_2$ of Jiao *et al.*[3] and are given by:

$$\mathbf{a_1} = \mathbf{e_2} = (2^{-\frac{1}{p}} - 2^{\frac{1}{2}}s)\mathbf{i} + (2^{-\frac{1}{p}} + 2^{\frac{1}{2}}s)\mathbf{j} \tag{1}$$

$$\mathbf{a_2} = \mathbf{e_1} - \mathbf{e_2} = (2^{-\frac{1}{p}} + 2^{\frac{1}{2}}s)\mathbf{i} + (2^{-\frac{1}{p}} - 2^{\frac{1}{2}}s)\mathbf{j} \tag{2}$$

where, $\mathbf{i}$ and $\mathbf{j}$ are the vectors that point to the superball faces along x and y, with length $d/2$, and s is found by solving and finding the smallest positive root:

$$\left|2^{-(1+\frac{1}{p})} - 2^{-\frac{1}{2}}s\right|^p + \left|2^{-(1+\frac{1}{p})} + 2^{-\frac{1}{2}}s\right|^p = 1 \tag{3}$$

and p is the shape parameter of the lattice site, as illustrated in Supplementary Figure 2a. For a change from $p = 2$ to $p \to \infty$, the two vectors describe the full transition from hexagonal to square symmetry. In order to avoid confusion between the lattice shape parameter p and the particle shape parameter m we refer to the lattice structures by the angle, α, via the direct relation with p shown in Supplementary Figure 2e. Supplementary Figure 2b shows the reciprocal-space basis vectors $\mathbf{b}_1$ and $\mathbf{b}_2$ with angle, β, that are obtained by converting the real-space vectors in the usual way[4]. To describe all the stacking sequences of layers with the same vectors we define the orientation of real space vector $\mathbf{a}_3$ perpendicular to vectors $\mathbf{a}_1$ and $\mathbf{a}_2$ as shown in Supplementary Figure 2c. Supplementary Figure 2d shows the obtained 3D reciprocal space lattice consisting of planes spanned by $\mathbf{b}_1$ and $\mathbf{b}_2$ that are spaced by $\mathbf{b}_3$.

The scattering *hkl* peaks are described by $\mathbf{q} = h\mathbf{b}_1 + k\mathbf{b}_2 + l\mathbf{b}_3$. We can now use the same *hkl* indices for all scattering peaks observed from the plastic FCC crystal and the two rhombohedral crystal structures.

Supplementary Figure 2f illustrates this effect for the relative positions of several q_{hk0} peaks with respect to the q_{110} peak for the full change in β. The latter peak was chosen as reference as it was observed in all 2D µrad-SAXS patterns of the different stacking sequences (Fig.2d, Fig.2e and Fig.4a). The *hkl* indices corresponding the scattering peaks present in a pure Face Centred Cubic (FCC) or Simple Cubic (SC) lattice are also indicated at $\beta = 60°$ and 90°, respectively.

From the monocrystalline 2D µrad-SAXS patterns β can be extracted in several different ways. Firstly, a direct extraction can be done by measuring the angle between the *h*00 and 0*k*0 peaks. Secondly, the change in β also changes the *q*-position of the peaks and their relative positions can be used to extract β, in a similar manner as in ref.5. Thirdly, the angle between the *hk*0 and the *11*0 peaks is also affected (Supplementary Figure 2g). We find good agreement between the three methods. However, the direct measurement of β from the 2D µrad-SAXS patterns is the most accurate and the other methods were only used when this analysis could not be applied due to missing *h*00 and 0*k*0 peaks.

Supplementary References

1 Rossi, L. *et al.* Cubic crystals from cubic colloids. *Soft Matter,* **7**, 4139-4142, doi:10.1039/c0sm01246g (2011).

2 Rossi, L. *et al.* Shape-sensitive crystallization in colloidal superball fluids. *PNAS,* **112**, 5286-5290, doi:10.1073/pnas.1415467112 (2015).

3 Jiao, Y., Stillinger, F. H. & Torquato, S. Optimal packings of superdisks and the role of symmetry. *Phys. Rev. Lett.,* **100**, 245504, doi:10.1103/PhysRevLett.100.245504 (2008).

4 Guinier, A. *X-ray Diffraction in Crystals, Imperfect Crystals, and Amorphous Bodies*. (Dover, 1994).

5 Zhang, Y., Lu, F., van der Lelie, D. & Gang, O. Continuous Phase Transformation in Nanocube Assemblies. *Phys. Rev. Lett.,* **107**, 135701, doi:10.1103/PhysRevLett.107.135701 (2011).
